# Attitude and potential benefits of modern information and communication technology use and telemedicine in cross-sectoral solid organ transplant care

**DOI:** 10.1038/s41598-021-88447-6

**Published:** 2021-04-27

**Authors:** Martin Holderried, Ansgar Hoeper, Friederike Holderried, Nils Heyne, Silvio Nadalin, Oliver Unger, Christian Ernst, Martina Guthoff

**Affiliations:** 1 eHealth Research-Group, Department of Strategic Medical Development and Quality Management, University of Tuebingen, Hoppe-Seyler-Str. 6, 72076 Tuebingen, Germany; 2 Institute for Healthcare and Public Management, University of Hohenheim, Hohenheim, Stuttgart, Germany; 3 Department of Diabetology, Endocrinology, Nephrology, Section of Nephrology and Hypertension, University of Tuebingen, Tuebingen, Germany; 4 Department of General-, Visceral- and Transplant Surgery, University of Tuebingen, Tuebingen, Germany; 5 Faculty of Business Management and Economics, Julius-Maximilians-University, Wuerzburg, Germany

**Keywords:** Hepatology, Health care, Nephrology

## Abstract

Situations like the COVID-19 pandemic urgently require the implementation of eHealth for vulnerable patient populations. Here we quantitatively evaluate use and potential of modern information and communication technology (ICT) in solid organ transplant (SOT) recipients. We conducted a structured, questionnaire-based, cross-sectional study that was addressed to patients after kidney, liver, pancreas, or combined transplantation. We focused on: sociodemographic data, present use of digital technologies in daily life and for health reasons, patients’ eHealth literacy, and their overall attitude towards eHealth. A total of 234 patients completed the questionnaire. Most of the patients (90%) have a web-enabled computer, 78.2% have a smartphone, and 71.8% regularly search the internet for health-related information. Sixty-eight percent would like to receive discharge summaries online, and 54% would like to chat online with their physicians. Even though ICT use in daily life was age-related, no significant difference could be shown for health reasons or the type of transplanted organ. Modern ICT use is predominantly accepted for health reasons by SOT recipients. Regardless of the transplanted organ, a deeper integration of eHealth has potential for improving cross-sectoral care. To successfully implement eHealth technologies in cross-sectoral care future research should include online physician–patient communication, data security, data safety, and the aspects of quality and safety of care.

## Introduction

Electronic Health (eHealth), which summarizes the use of modern information and communication technology (ICT) in healthcare^1^, has become a promising tool to improve and facilitate patients’ attendance and motivation in health care^2^. The general use of modern ICT has markedly increased in recent years^3^, with internet penetration in the United States (US) reaching 89.4% (and 87.7% in Europe)^4^. A particular challenge in Germany, where the study was carried out, is the limited adoption of eHealth because of data security and data privacy concerns, although recent changes in legislation will facilitate its uptake With the increased use of ICT in daily life, novel possibilities to access patients interactively arise by implementing eHealth and mobile Health (mHealth) applications in patients’ care, follow-up, and adherence. Of note, the current COVID-19 pandemic rises the need for urgent implementation of eHealth for sensitive patient populations.

Previous studies in various medical fields have shown that the use and attitude towards online health-related information search depend on geographic, sociodemographic, educational, socioeconomic aspects, and health status^5–9^.

Published literature on eHealth and modern ICT use in solid organ transplantation remains scarce but has been growing in recent years^10^. A study in Belgium showed that only 28% of patients that had undergone solid organ transplantation owned a smartphone, whereas 72% were able to use the internet in daily life^11^. Some projects have aimed to introduce telemedicine in follow-up care^12–14^, and others have addressed education for living kidney donation^15^. Schmid and colleagues developed a telemedical-supported case management tool for the first year after kidney transplantation, which led to markedly increased adherence compared to standard follow-up^14^. These authors were able to show that, by this approach, the healthcare costs could be substantially reduced^16^. Other studies concentrated on single aspects, such as website information^17^ or mobile phone use^18,19^.

In solid organ transplantation, especially in pediatrics and in kidney transplantation, adherence is more and more recognized as a key factor of long term allograft survival^20,21^. Adherence is defined as “the extent to which a person’s behavior—taking medication, following a diet, and/or executing lifestyle changes, corresponds with agreed recommendations from a health care provider”^22^. The importance of adherence after kidney transplantation has been emphasized in recent years, as chronic antibody-mediated rejection, a consequence of under immunosuppression, has emerged as a major cause of allograft loss^23,24^. Nevertheless, only about 70% of patients after kidney transplantation are adherent^25,26^; therefore, further methods to increase adherence are urgently needed, and innovative eHealth applications might be interesting candidates for the task.

As a basis for the development of specific eHealth tools in solid organ transplantation, both physicians and software/app developers require a thorough knowledge of the influencing factors in this patient population. Nevertheless, there is little understanding about the present ICT use and the patients’ perspectives towards a deeper integration of eHealth in cross-sectoral care of solid organ transplant patients. The objective of the present study was to close this information gap. We assessed the current use of modern ICT in private life and for health reasons in kidney, pancreas, and liver transplant recipients. We further evaluated the potential and influencing factors of patients’ attitudes and willingness for the use of eHealth to improve cross-sectoral patient care. Our data provide a solid basis for further development and scientific investigation of specific and adapted comprehensive and cross-sectoral telecare models with suitable eHealth applications for this patient population.

## Methods

Our quantitative study was conducted by the delivery of a structured questionnaire addressed to patients after kidney, liver, pancreas, or combined solid organ transplantation. The contact data used were obtained from the database of the Tuebingen University Hospital Collaborative Transplant Center, Germany. Patients or the public were not involved in the design, or conduct, or reporting, or dissemination plans of our research.

### Ethics

This survey-based study was approved by the Institutional Ethics Committee of the Medical Faculty and University Hospital of Tuebingen (740/2016BO2) and conducted in accordance with the declaration of Helsinki.

### Patients

Patients from the post-transplant follow-up care after kidney, liver, pancreas, or combined organ transplantation were invited to participate in the study. Time since transplantation ranged from one to 33 years. All patients were contacted by phone. After obtaining the patients’ informed consent, the questionnaire was sent to patients by mail. Following recommendations in the literature^27^, the study sample was generated using a systematic sampling technique. Each patient was ≥ 18 years old. Within 6 months, a total of 284 transplant patients had been asked to participate in the survey-based study. For statistical analysis, the patients were divided into two age groups of < 55 and ≥ 55 years, according to the approximate median age of patients at time of kidney transplantation in the Eurotransplant area^28^.

### Questionnaire

Our questionnaire was developed by an interprofessional team of physicians specialized in organ transplant surgery and transplant nephrology, eHealth specialists, quality managers, and public health researchers, based on the current literature and their own experience with pilot studies in other medical fields^7,9,29–31^. The structured questionnaire included the following aspects: sociodemographic data (e.g., education level, and insurance status); the present use of modern ICT in private life and for health reasons (e.g., social network membership, smartwatch possession, online information search); online health-related information search; and attitude and willingness to use eHealth applications for cross-sectoral care (e.g., willingness to use online health messaging or video visits).

**Figure 1:**
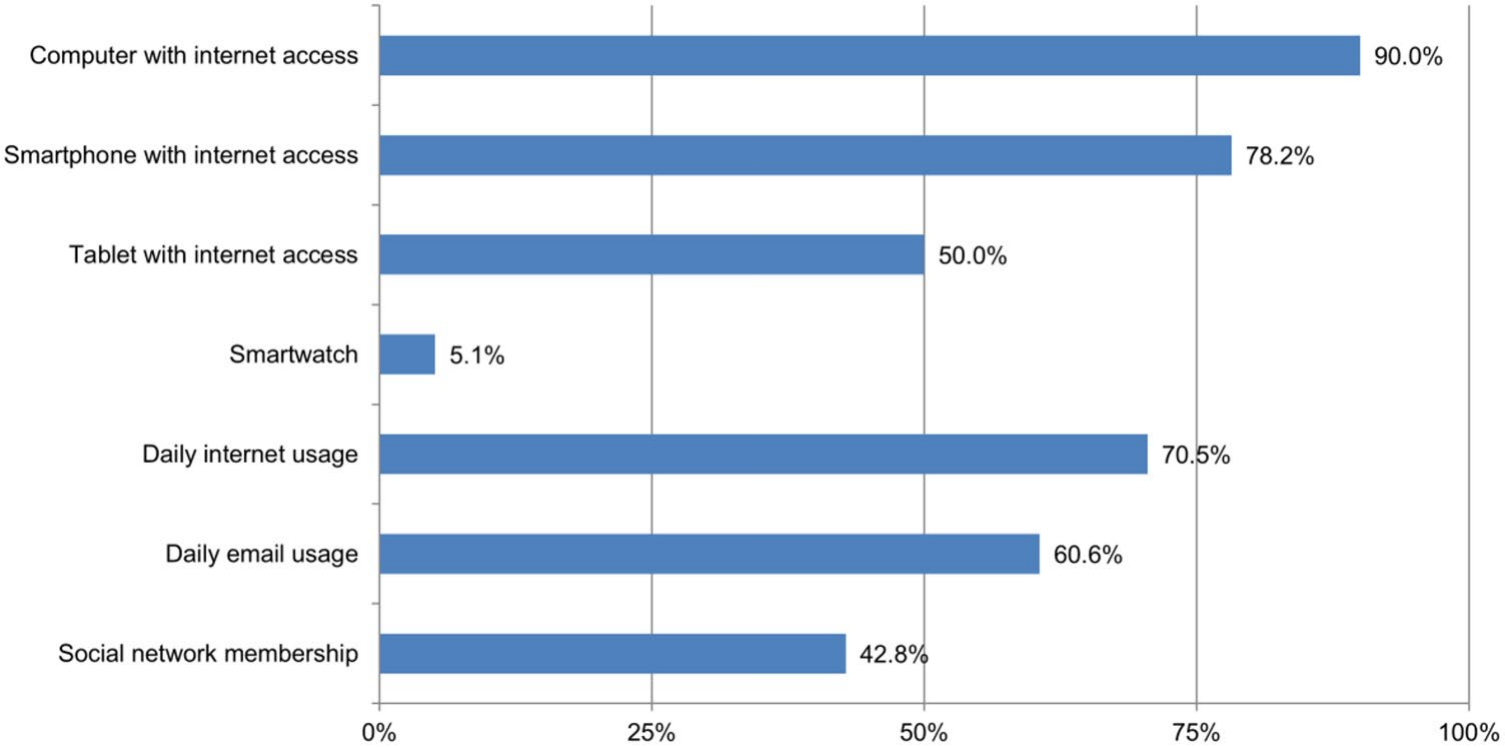
Penetration and present use of modern ICT in solid organ transplant recipients in % (answers given by the 234 responders).

**Figure 2:**
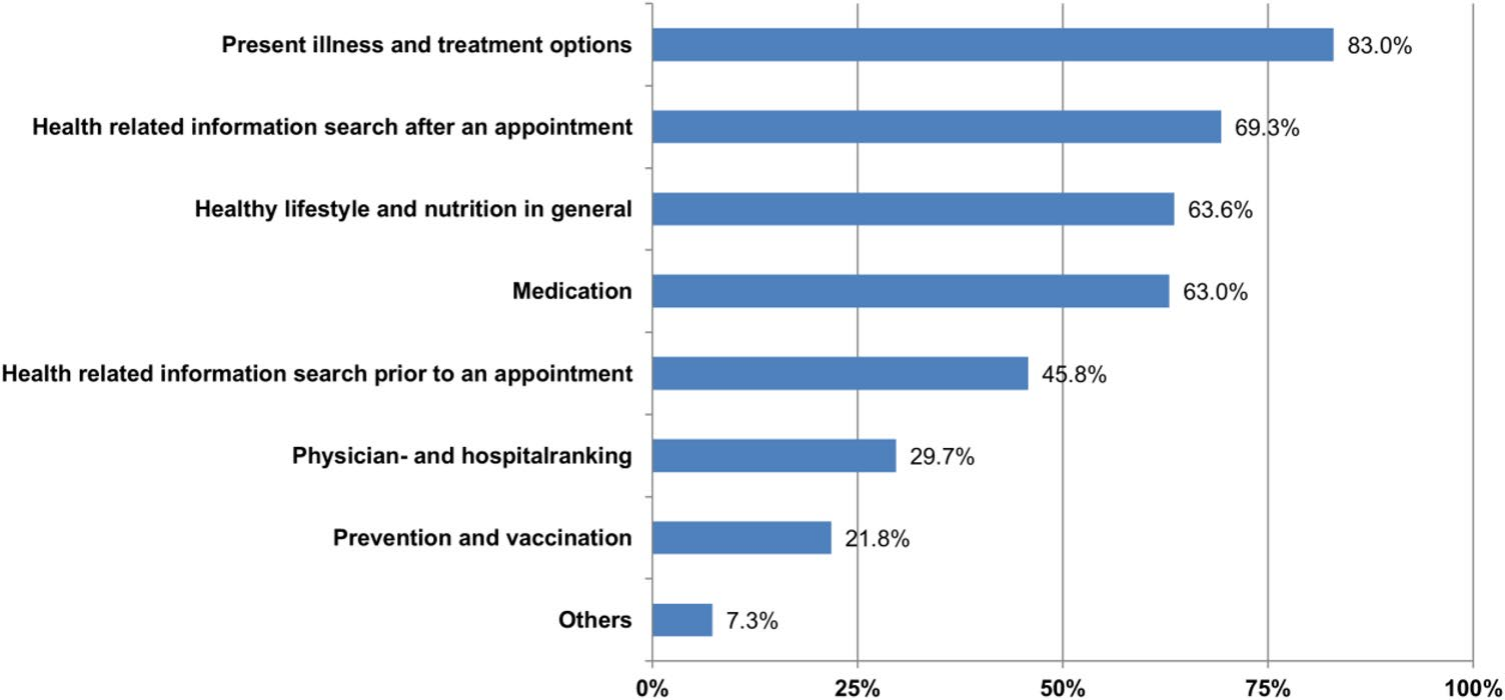
Online health related information search in solid organ transplant recipients in % (answers given by the 234 responders).

**Figure 3:**
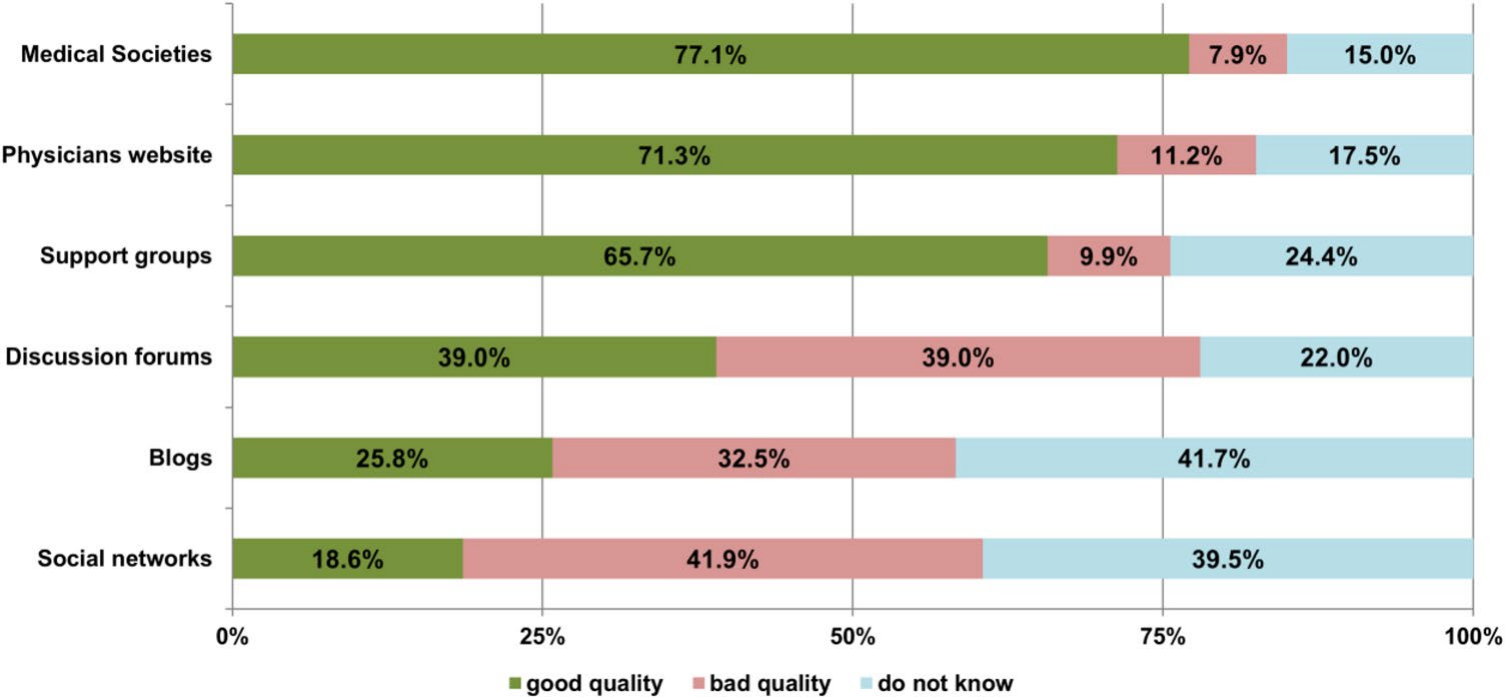
The patients` assessment of the quality of online sources for health related information in % (answers given by the 234 responders).

**Figure 4:**
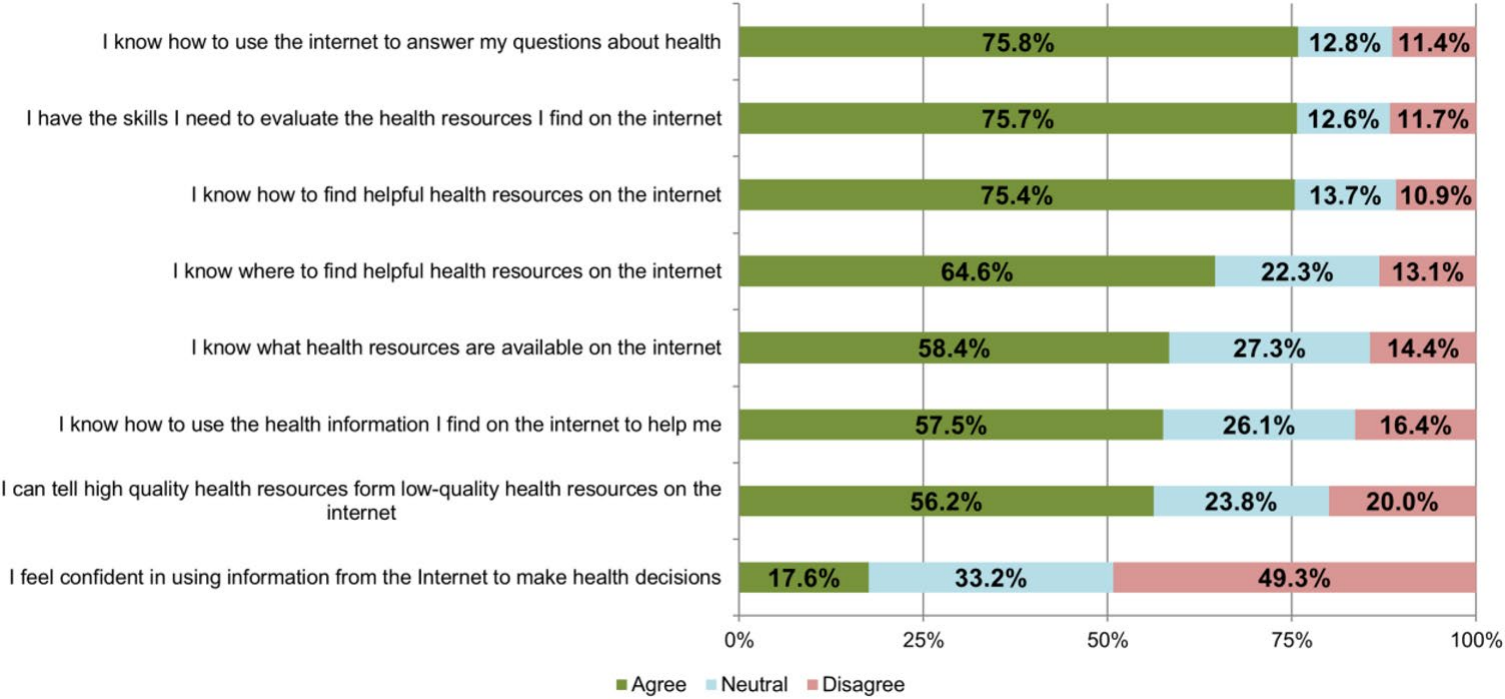
Results of the eHealth Literacy Scale (eHEALS) in % (answers given by the 234 responders).

Structurally, the questionnaire consisted of closed-ended questions (e.g., smartwatch possession, online health-related information search before and after medical consultation) and evaluation scales for specific measures (e.g., online communication of general and personal health-related information). The surveyed aspects, including the number of statements and scale scores for each response, are summarized in detail in Tables 1 and 2 and Figs. 1, 2, 3, 4.

**Table 1:** Age-related characteristics of the study sample (answers given by the 234 responders).

	Total	Age		*p*-Value
	n (%)	< 55 n (%)	≥ 55 n (%)	
**Gender**				
Female	112 (48.1)	60 (53.6)	52 (46.4)	
Male	121 (51.9)	64 (52.9)	57 (47.1)	n.s. (0.917)
**Community size (inhabitants)**				
< 2000	38 (16.6)	22 (57.9)	16 (42.1)	
2000–30,000	137 (59.8)	73 (53.3)	64 (46.7)	
> 30,000	54 (23.6)	26 (48.1)	28 (51.9)	n.s. (0.645)
**Education level**				
Low	81 (35.4)	41 (50.6)	40 (49.4)	
Middle	66 (28.8)	35 (53.0)	31 (47.0)	
High	82 (35.8)	46 (56.1)	36 (43.9)	n.s. (0.781)
**Employed**				
No	108 (46.2)	34 (31.5)	74 (68.5)	
Yes	126 (53.8)	91 (72.2)	35 (27.8)	** < 0.001**
**Frequency of medical consultation in the last year**				
≤ 10 times	96 (41.2)	52 (54.2)	44 (45.8)	
> 10 times	137 (58.8)	72 (52.6)	65 (47.4)	n.s. (0.808)
**Missed appointments in the past**				
No	168 (71.8)	80 (47.6)	88 (52.4)	
Yes	66 (28.2)	45 (68.2)	21 (31.8)	**0.005**
**Insurance status**				
Statutory health insurance	194 (82.9)	106 (54.6)	88 (45.4)	
Private health insurance	40 (17.1)	19 (47.5)	21 (52.5)	n.s. (0.410)
**Medication intake**				
≤ 5 different medications/day	97 (41.8)	63 (64.9)	34 (35.1)	
> 5 different medications/day	135 (58.2)	61 (45.2)	74 (54.8)	**0.003**
**Transplanted organ (multiple answers possible)**				
Liver	64 (27.4)	33 (51.6)	31 (48.4)	
Kidney	174 (74.4)	93 (53.4)	81 (46.6)	
Pancreas	32 (13.7)	17 (53.1)	15 (46.9)	
**Daily measurement of blood pressure**				
No	112 (48.7)	63 (56.3)	49 (43.8)	
Yes	118 (51.3)	58 (49.2)	60 (50.8)	n.s. (0.281)
**Daily measurement of personal weight**				
No	110 (47.8)	63 (57.3)	47 (42.7)	
Yes	120 (52.2)	58 (48.3)	62 (51.7)	n.s. (0.175)

Bold values are statistcally significant for p<0.05 values.

**Table 2:** Association between gender, age, education level, missed appointments, community size, modern ICT use in daily life and the attitude towards ICT use for health reasons.

	Present ICT use in daily life	Attitude towards ICT use for health reasons
	OR [95% CI]	OR [95% CI]
**Gender**		
Female	1	1
Male	1.46 [0.76–2.78]	0.92 [0.49–1.73]
**Age**		
< 55	1	1
≥ 55	**0.50 [0.26–0.96]**	0.69 [0.37–1.29]
**Education level**		
Low	1	1
Middle	1.73 [0.79–3.78]	1.66 [0.80–3.44]
High	**2.64 [1.21–5.75]**	**4.70 [2.11–10.45]**
**Missed appointment**		
No	1	1
Yes	1.99 [0.88–4.52]	1.82 [0.85–3.88]
**Community size**		
Low	1	1
Middle	1.31 [0.57–3.04]	1.39 [0.60–3.19]
High	1.48 [0.52–4.12]	1.32 [0.48–3.61]

Bold values are statistcally significant for p<0.05 values.

To evaluate the perceived knowledge and skills for using digital information technology for health reasons among the study population, the 8-item based eHealth literacy scale (eHEALS) was included in the questionnaire^32,33^.

### Statistical analysis

We first performed a descriptive analysis to get an overview of the answers to the study-specific items. Bivariate analysis was used to investigate the relationships between the sociodemographic aspects of the study population, the present use of modern ICT, the transplanted organ, as well as the attitude towards eHealth for further use in cross-sectoral care. To analyze statistically significant trends, the surveyed statements about the eHealth potentials were transformed from a four-point Likert scale to binary response variables. “Fully” and “fairly” were rated as positive and “not at all” and “rather not” were rated as negative. The same procedure was used with the 5-point Likert responses to the eHealth literacy scale: To create the data set for these bivariate statistical calculations, the statements were transformed to the following response variables: positive (fully, fairly), negative (rather not, not at all), and neutral.

To assess the differences in the relative frequencies between the age groups, cross-tabulation and Pearson’s chi-square tests were used. The study-specific results were presented as numbers, percentages, and two-tailed *p*-values.

To examine the relationship between gender, age, education level, community size, percentage of patients who missed appointments in the past (as an indicator for medical adherence), modern ICT use and the attitude towards ICT use for health reasons, we used multivariate logistic regression. The results are expressed as odds ratios (ORs) with 95% confidence intervals (CIs).

Non-responders to specific survey questions were excluded from the analyses of those questions. For all analyses performed in our study, p-values ≤ 0.05 were considered as statistically significant. The IBM Statistical Package for the Social Sciences (SPSS) Version 25 (IBM Corp., Armonk, NY, USA) was used for all statistical analyses.

## Results

A total of 284 transplant patients were asked to participate in the survey-based study. 248 patients replied to the survey and 234 of them returned a completed questionnaire. This represents an overall response rate of 82.4% (234/284) and a completion rate of 94.4% (234/248). Both rates are extremely high for this kind of survey-based study. Consequently, the high rates allow for a robust statistical analysis of the study sample^27^.

### Patient characteristics

Of 234 patients with completed questionnaire, 48.1% were female, and 51.9% male; 53.4% of patients were < 55 years old, and 46.6% were ≥ 55 years old. Younger patients were significantly more employed and had to take significantly fewer medications per day (*p* = 0.003). Furthermore, the number of medical appointments was significantly positively related to an older age, the percentage of patients who missed appointments in the past was negatively related to an older age. In all other aspects, age did not differ significantly. The age-related sociodemographic factors and further patient characteristics are given in Table 1.

### Penetration and use of modern ICT in daily life

Overall, the penetration and use of modern ICT in solid organ transplant recipients were high. Only the use of smartwatches (with a penetration of 5.1%) was still in its infancy (Fig. 1).

The penetration of smartphones (78.2%) and tablets (50%) was significantly higher in patients < 55 years old (*p* < 0.001 and *p* = 0.008, respectively).

Daily internet and email use, as well as social network membership, also showed a significant age relation (all *p* ≤ 0.045), dominated by the younger study participants. Furthermore, occupation, higher education, and the percentage of patients who missed appointments in the past were significantly positively associated with the use of modern ICT in daily life (all *p* ≤ 0.024).

### Online health-related information search

Overall, online health-related information search was important for solid organ transplant recipients. Information search about present illness and treatment options showed the highest priority with 83.0%, followed by health-related information search after medical appointments (69.3%) and information search about healthy lifestyle and nutrition in general (63.6%). Further health-related topics of interest for the study population are shown in detail in Fig. 2. Here, the data showed no significant relation to age, gender, or occupation. Also, education was only positively associated with information search about healthy lifestyles and nutrition (*p* = 0.013).

Regarding the kind of online information sources, the surveyed solid organ transplant patients most often used the information of medical societies (62.3%), followed by physicians’ websites (50.0%), discussion forums (38.9%), and support groups (34.0%). Blogs (14.2%) and social networks (12.3%) were ranked lowest.

Analyzing the mentioned online information sources for health-related information, no difference in use could be shown for gender, age, or education level. Only the use of medical societies was significantly positively associated with higher education (*p* = 0.008).

The patients’ assessments of the quality of the mentioned online health-related information sources are shown in Fig. 3. Here, gender showed no significant impact on the rating. Also, age, education level, and occupation showed no significant influence, except that younger and employed patients rated the quality of online information by support groups higher (all *p* ≤ 0.015). In addition, low-educated patients rated the quality of social media information higher than high-educated patients (*p* = 0.045).

### eHealth literacy scale (eHEALS)

Regarding the participants’ comfort and skills in using digital information and communication technology for health reasons, more than three quarters responded that they knew how to use and how to evaluate the online information resources for health reasons. Nevertheless, even if they were confident to tell high-quality from low-quality information and knew how to use this information, only a few patients (17.6%) felt confident in using information from the internet to make health decisions. The detailed results of the 8-item eHEALS are given in Fig. 4.

### Attitude and willingness to use eHealth applications for cross-sectoral care

Overall, the study participants reported a positive attitude towards the further use of eHealth technologies for cross-sectoral transplant patient care. Two-thirds (66.8%) would like to schedule their appointments online, and 68.9% would like to get automated appointment reminders online. Most would find it helpful to receive general online information prior to a medical appointment (65.9%), would like to receive their medication plan online (68.9%), would like to get test results (66.5%), and would like to get discharge summaries by email (67.6%). In order to better plan and prepare an inpatient treatment prior to admission, 56.9% of the patients would use a hospital app.

Among the patients, 31.8% reported that they would like to have online video visits with their physicians, and 53.8% would chat online with them, both with no significance regarding age, education level, or occupation. Video visits were significantly more desired by male patients (*p* = 0.010). Most (67.8%) of the questioned transplant recipients would use a personal electronic health record (PEHR) that they can manage themselves. Here, no significance regarding the above-mentioned patient characteristics could be shown. 38.7% are convinced that further online communication in cross-sectoral care could have a positive impact on physician–patient contact, and 45.7% assumed a positive impact of eHealth use for the overall treatment quality. This potential was especially acknowledged by younger and occupied patients, as well as by patients with higher education and private health insurance (all *p* ≤ 0.038).

Data security aspects have also been rated very important by the patients. Almost half (47.0%) of the participants reported concerns about data security by further use of modern ICT in cross-sectoral care, which again did not show a significant influence of the above-mentioned patient characteristics.

Multiple logistic regression analysis showed neither a significant difference in present ICT use in daily life, nor in the attitude towards ICT use for health reasons, regarding gender and community size. Present ICT use in general was significantly higher in younger patients and patients with a higher level of education. The results of the multiple logistic regression analysis are shown in detail in Table 2.

## Discussion

Digitalization awareness and modern ICT use are growing rapidly in various fields, especially in the healthcare sector. Online communication with friends, relatives, and for business purposes has become an integral part of everyday life, and current statistics show that internet use continues to increase significantly in daily life, regardless of age^4^. Also, in healthcare, the potential of modern ICT use for the improvement of cross-sectoral care seems to be enormous, and eHealth is, therefore, getting increasing attention as a focus of public, political, and healthcare agendas^34–36^. Despite its unquestionable potential, especially in this patient population, little is known about the perspectives regarding the penetration and use of modern ICT in everyday life and for health reasons. In particular, there is a need to better understand these patients’ attitudes and influencing factors of the different aspects of eHealth use for online communication in cross-sectoral care and online health related information search. Therefore, we conducted our study with a special focus on the different aspects and factors that influence the penetration and use of digital ICT for everyday life and for health reasons in solid organ transplant patients.

The gender ratio of the study population (female:male = 1:1.08) was close to the general population in Germany (female:male = 1:0.97), where the study was done, and also similar to the population of the US (female:male = 1:0.97)^37^.

Regarding age, the questioned patient group was slightly older (53.4% < 55 years and 46.6% ≥ 55 years) than the general population in Germany (62.68% < 55 years and 37.32% ≥ 55 years) and the United States (71.03% < 55 years and 28.97% ≥ 55 years)^37^. The older age reflects the high presence of kidney transplant recipients (74.4%) in the study population: A growing number of patients aged > 50 years are on the kidney transplant waiting list in the US^38^, and almost 53% of patients who received kidney transplantation in Europe in 2016 were > 55 years old^39^.

Despite our older survey population, the penetration of ICT use in daily life within the study population was high. Most (78%) reported having a smartphone, a much higher number than was reported in a recent Belgian study (only 27.9%)^11^. Ninety percent of the patients were in possession of a computer with internet access, but interestingly, only 70.5% used the internet on a daily basis. Even if this number is below the general internet use in Germany (96.2%)^5^, it is comparable to studies in other medical fields^29^. Our finding that 42.8% of the questioned patients reported a social network membership shows the potential for expanding the use of online support groups for transplant patients and their families, although this must take the high demands on data privacy and data security into account. Even if the smartwatch use at present is very low within this patient population, this aspect should be tracked continuously. We believe that the penetration of these devices will increase rapidly in everyday life, so that smartwatches can be integrated into mobile health use in the near future.

Regarding the sociodemographic factors, gender did not significantly affect internet use in daily life. However, age and occupation of the transplant recipients had a significant impact on the use of modern ICT in everyday life, which is consistent with previous studies in other medical fields^5,6,9,31^. It is noteworthy that the overall high penetration rate of mobile digital device in our study was significantly associated with younger age. This is important information for further investigation and development of eHealth applications, especially for supporting the self-management and the mobile communication of personal medical information with solid organ transplant recipients. In contrast to previous studies, we could find no overall significance for health-related internet use regarding age, education, or occupation^5,6,9,31^.

Online health-related information search is regarded as an instrument to empower patients. Our study population showed a high penetration of online health information seeking (OHIS) with no association to age, gender, or occupation, which is in contrast to a previous study within the German population^8^. The main reason for OHIS in solid organ transplant patients is especially related to the present illness of a chronic condition, which goes along with OHIS outside the field of solid organ transplantation^40^. In comparison to the results of earlier studies done with a lower OHIS rate^5^, our results support the hypothesis that OHIS is becoming increasingly important and will play a central role for patient education and empowerment in the near future.

Regarding the attitude towards eHealth, about two-thirds of the solid organ transplant recipients would use online appointment scheduling and exchange general and personal medical information online. Also, more than half of the patients rated online health messaging services as useful for cross-sectoral communication of medical information. About one-third of the transplant recipients are willing to use real-time video communication with their healthcare providers. The willingness to use a personal electronic health record (PEHR) was rated much higher, interestingly, with no significance regarding gender, age, education, or occupation. Nevertheless, although half of the patients responded that eHealth use would improve treatment quality, they also had concerns about data security aspects regarding the online communication of health-related information. This highlights the great potential of eHealth to improve cross-sectoral care but also the need for high-level standards of data security and privacy, not only from the point of view of health authorities, but, importantly, also from the patients’ perspective. In the cohort of solid organ transplant recipients, the greatest benefit of integration of eHealth in cross-sectoral care is the aspect of adherence, especially of adherence to medication. Non-adherence to medication is a major cause of allograft loss in kidney transplantation^25^ and is also an important issue in liver transplantation^41^. Increasing adherence is, therefore, one of the major goals of transplantation medicine to improve long-term allograft survival^42^. Recognizing that measures to improve adherence are more effective when they are multidimensional^42–45^, modern ICT, and in particular mobile device use in the context of cross-sectoral care and self-management after solid organ transplantation, have the potential to markedly increase patients’ adherence.

It is important not to overstrain patients with modern ICT tools. Older patients, in particular, might have difficulties handling these modern technologies, so for eHealth applications, ease of use will be a key success factor. Furthermore, education had a significant impact on the use and attitude towards eHealth in our study and needs to be taken into account. Even though not investigated in our study, the authors assume that it would be appropriate to test new forms of patient education, such as easily accessible short videos. Based on our study, the aspects of data security, data privacy, and data availability are very important aspects for further eHealth strategies, even if data availability was not particularly addressed in the present study. From the authors' point of view, these aspects, in addition to the ease of use and technical interoperability of the eHealth systems, create essential prerequisites for improving patient engagement, quality, and safety, as well as the efficiency of cross-sectoral patient care. Furthermore, this enables the integration of machine learning and artificial intelligence for further improvement of precision medicine in the field of solid organ transplantation.

Our study does have limitations: It is a single center study of a western European country and therefore not generalizable for all other countries. Furthermore, we do not present active eHealth interventions; however, our study provides unique data for further development of eHealth strategies for cross-sectoral care of this chronic patient population.

## Conclusion

In our study, we could demonstrate a high penetration, acceptance, and positive attitude towards eHealth in solid organ transplant recipients, forming a basis for the further development of comprehensive cross-sectoral (tele)care platforms with specific eHealth applications for this patient population. It is imperative that healthcare providers are not only open to digital development but actively contribute to the establishment of this disruptive innovation. Especially in times we are facing now with the COVID-19 pandemic, we need rapid implementation of telemedicine for immunocompromised patients such as solid organ transplant recipients.

## Data Availability

All data are available from the following public repository: www.kaggle.com/dataset/bb0a38b0f0a3c1a938019e4b983c6c5a307319eebf6bcd666212f02f74666165
